# Low Protein-High Carbohydrate Diets Alter Energy Balance, Gut Microbiota Composition and Blood Metabolomics Profile in Young Pigs

**DOI:** 10.1038/s41598-020-60150-y

**Published:** 2020-02-24

**Authors:** Shelby Spring, Hasitha Premathilake, Udaya DeSilva, Cedrick Shili, Scott Carter, Adel Pezeshki

**Affiliations:** Department of Animal and Food Sciences, Oklahoma State University, Stillwater, OK 74078 USA

**Keywords:** Homeostasis, Molecular medicine

## Abstract

Reducing dietary crude protein (CP) beyond a certain threshold leads to poor growth performance in pigs; however, the underlying mechanisms are not well understood. Following an adaption period, thirty-seven weaned pigs were weight matched (8.41 ± 0.14 kg), housed individually and randomly assigned into three groups with different dietary CP levels: 24% CP (CON; n = 12), 18% CP (n = 12) and 12% CP (n = 13) for 28 days. The body weight was not different between the CON and 18% CP diets, but 12% CP significantly decreased body weight after day 21. Compared to the CON, pigs fed with 12% CP decreased feed intake day 17 onwards. The 12% CP diet increased the energy expenditure during week 1 compared to the CON. The 12% CP influenced starch and sucrose, nitrogen, and branched-chain amino acids metabolism pathways. The feces of pigs fed with 12% CP were less enriched in *Prevotella*, but had higher relative abundance of *Christensenedilaceae*, *Aligiphilus* and *Algoriphagus* than CON and 18% CP. Overall, reducing dietary CP by 50%, but not by 25%, significantly influenced the physiological responses in nursery pigs. The pigs fed with low or standard protein diets had differential bacterial communities in their feces as well as serum metabolomics profile.

## Introduction

Modern pig production is criticized due to environmental concerns associated with using high dietary protein level resulting in excessive nitrogen excretion^1^. Emissions of ammonia from swine manure can contribute to eutrophication and acidification of sensitive ecosystems^1^ and can have adverse effects on human health^2^. Beside the e nvironmental pollutions and waste of protein in the current global shortage of protein for livestock^3^, the high protein diets are associated with increased diet cost, anti-nutritional factors affecting the gut integrity^4^ and incidence of diarrhea in weaned pigs^5^. A significant decrease in nitrogen excretion has been reported in pigs received slightly low protein diets, *i.e*. diets with 25% reduced crude protein (CP), supplemented with essential amino acids^6,7^. These diets improve the body weight and growth performance or have no negative impact on performance and feed efficiency of young and growing-finishing pigs^6,8–10^. Moderate to severe reduction in dietary protein (>25% reduction) may produce more beneficial results in terms of nitrogen excretion; however, these diets lead to poor performance in growing pigs and lactating sows even when supplemented with limiting amino acids^11–15^. The underlying factors that regulate the performance of nursery pigs fed with moderately low protein diets is less known. Understanding the mechanisms by which these diets reduce the growth performance may lead to development of dietary strategies and nutraceutical products that not only are environment-friendly, but also have no negative impact on growth performance of pigs so that can encourage commercial swine producers  to apply those strategies and products.

Consistent with data from rodent studies^16–22^ and the protein leverage hypothesis stating that several animal species give priority to meet their protein needs over other dietary components^23^, severe reduction in dietary protein increases the feed intake as well as heat production in young pigs^24–26^. A pioneer study^24^ reported a higher energy intake and suggested an increased energy expenditure (EE) in pigs fed with extremely low protein diets (3% CP). Further, another study^27^ reported that protein deficient pigs consuming high or low-calorie diets maintained equal body weight suggestive of increased EE in these pigs. In support of these studies, others^25^ reported an increased feed intake and EE in growing pigs fed with extremely low protein diets (2.38% CP). In these studies, limited numbers of pigs were used to assess the effect of dietary protein level on energy balance. Unlike severe reduction in dietary protein, moderately low protein diets (12–15% CP) supplemented with essential amino acids either had no effect^28,29^ or decreased the EE in growing and finishing pigs^10,30,31^. However, all of these studies measured the EE acutely towards the end of their studies and hence the temporal effect of diets in earlier weeks of studies was not captured.

In addition to energy balance factors (*i.e*. feed intake and EE), gut microbiota may significantly contribute to metabolic responses of young pigs to low protein diets through its important role in nutrient metabolism^32^. Dietary proteins and carbohydrates are considered as major dietary factors regulating the gut microbiota composition in rodents^33^. Low protein and high carbohydrate diets have been reported to increase Firmicutes-to-Bacteroidetes ratio in cecum^34^. Although the effect of diets with 25–35% reduced protein content on the communities of intestinal bacteria has been previously studied in weaned pigs^35,36^, there are limited data on the gut microbiota composition of young pigs fed with diets with 50% reduced protein content. While the impact of age on the composition of gut microbiome in pigs is well documented^37^, the effect of moderately low protein diets (10–13% CP) supplemented with essential amino acids on intestinal microbiota has been only assessed in finishing and growing pigs^38,39^. The large intestine bacterial population utilizes the nutrients with lower digestibility including some proteins and produce metabolites such as amines and short chain fatty acids^40^. The metabolomic profile of hind gut in growing pigs fed with different levels of dietary protein has been previously reported^39^, but little is known on blood metabolomic profile of weaned pigs fed with low protein-high carbohydrate diets. Therefore, the objective of this study was to determine the effect of low protein-high carbohydrates diets on energy balance, blood metabolomics profile and fecal microbiota composition in weaned pigs.

## Results

### Feed intake, body weight, energy expenditure and respiratory quotient

**Figure 1:**
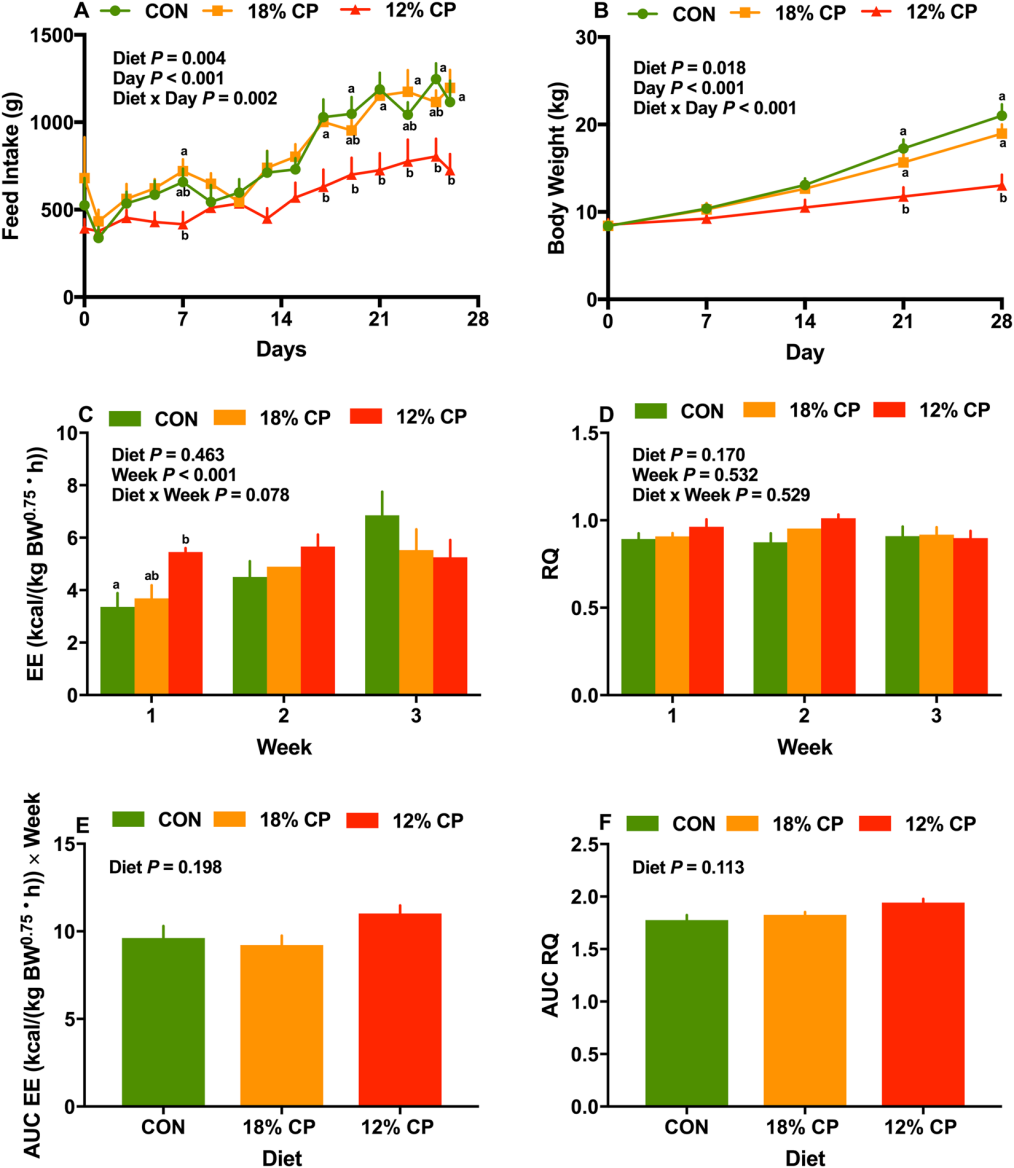
The effect of low protein diets on energy balance. (**A**) feed intake, (**B**) body weight, (**C**) mean energy expenditure (EE), (**D**) mean respiratory quotient (RQ), (**E**) area under curve (AUC) for EE, (**F**) AUC for RQ. CON, control diet with 24% crude protein (CP); 18% CP, low protein diet with 18% CP; 12% CP, low protein diet with 12% CP. Among groups, values with different superscripts are significantly different (P < 0.05). The values are means ± standard errors of means. n = 12, n = 12, n = 13 for CON, 18% CP and 12% CP groups, respectively for feed intake and body weight data; n = 7, n = 7, n = 8 for CON, 18% CP and 12% CP groups, respectively for EE and RQ data.

Overall, the effect of diet, day and the interaction of diet and day on daily feed intake were significant (P = 0.004, P < 0.001, P = 0.002, respectively; Fig. 1A). No significant difference in daily feed intake was detected when CON and 18% CP groups were compared (Fig. 1A). Compared to 18% CP, 12% CP had a tendency for decreased feed intake after day 14 (P = 0.08). Furthermore, compared to CON and 18% CP, pigs fed with 12% CP exhibited a significant decrease in feed intake on day 17 onwards (Fig. 1A). During the week 1 and 2, there was no difference in cumulative weekly feed intake among dietary treatments (Supplementary Fig. S1A). The cumulative weekly feed intake for 12% CP was significantly lower than CON and 18% CP on week 3 (P < 0.011; Supplementary Fig. S1A). During week 3 and 4, there was no difference in weekly feed intake comparing CON with 18% CP. Pigs fed 12% CP had a tendency for decreased feed intake on week 4 compared to both CON and 18% CP pigs (P < 0.074; Supplementary Fig. S1A).

Initial body weight among dietary groups was not different (P = 0.978; Table 1). Overall, the effect of diet, day and the interaction of diet and day for body weight were significant (P = 0.018; P < 0.001; P < 0.001; Fig. 1B). The final body weight of pigs in 12% CP group was 38% lower than CON and 31% lower than 18% CP (Table 1). Compared to CON and 18% CP, the body weight of the pigs fed with 12% CP was significantly decreased by day 21 onwards (Fig. 1B). No differences in body weight were detected between CON and 18% CP groups (Fig. 1B). Throughout the study, 12% CP significantly decreased weekly body weight gain compared to CON and 18% CP (Supplementary Fig. S1B). Except week 3, body weight gain for 18% CP was not different from CON (Supplementary Fig. S1B).

**Table 1:** Effect of dietary protein content on growth measurements.

Measurements	CON^1^	18% CP^1^	12% CP^1^	SEM^2^	P-value
Initial body weight, kg	8.40	8.40	8.50	0.70	0.978
Final body weight, kg	21.02^a^	18.97^a^	13.04^b^	1.95	<0.001
ADG^3^, g/d	485.87^a^	406.41^a^	173.41^b^	27.53	<0.001
ADFI^3^, g/d	837.20^a^	839.53^a^	591.60^b^	28.88	0.007
G:F^3^, g/g	0.58^a^	0.48^a^	0.29^b^	0.04	<0.001
ADPI^3^, g/d	191.88^a^	164.71^a^	67.44^b^	0.15	<0.001
G:P^3^, g/g	2.55	2.48	2.57	0.14	0.334

^1^CON, control diet with 24% crude protein (CP); 18% CP, low protein diet with 18% CP; 12% CP, low protein diet with 12% CP.

^2^SEM: standard errors of means.

^3^ADG: average daily gain; ADFI: average daily feed intake; G:F: gain:feed ratio; ADPI: average daily protein intake; G:P: gain:protein ratio.

^a,b^Within a row, values with different superscripts are different (P < 0.05).

Overall, the effect of diet on average daily gain (ADG), average daily feed intake (ADFI), gain:feed ratio (G:F) and average daily protein intake (ADPI) was significant (P < 0.01; Table 1) with lower ADG, ADFI, G:F and ADPI for 12% CP than 18% CP and CON. No differences in gain:protein ratio (G:P) was detected among groups (Table 1). There was no difference in final body weight, ADG, ADFI, ADPI and G:F ratio, when comparing CON and 18% CP (Table 1). The effect of diet on weekly G:F was significant, while the effect of week and the interaction of diet and week on G:F was not significant (P < 0.001; P = 0.716; P = 0.737; Supplementary Fig. S1C). The 12% CP decreased G:F throughout the 4 week compared to CON and 18% CP (Supplementary Fig. S1C). Except week 3, the G:F for 18% CP was not different compared to CON (Supplementary Fig. S1C). The effect of diet, week and the interaction of diet and week for weekly G:P was not significantly different among dietary treatments (Supplementary Fig. S1D).

Overall, mean weekly EE was not significant among dietary groups, while the effect of the week was significant and the interaction of diet and week tended to be significant (P = 0.463; P < 0.001; P = 0.078 respectively; Fig. 1C). Pigs fed with 12% CP increased the EE by 62% during week 1 compared to the CON diet (P = 0.017; Fig. 1C). The mean weekly EE for 18% CP was not different from CON and the 12% CP on week 1 (Fig. 1C). There was no difference in mean weekly EE across diets on week 2 and week 3 (Fig. 1C). Mean weekly respiratory quotient (RQ) was not different among diets throughout the experiment (Fig. 1D). When EE or RQ data were expressed as area under the curve (AUC), there was no significant difference among diets (Fig. 1E,F).

### Blood cytokines

No statistical differences were detected across groups for serum concentration of interleukin 8 (IL-8), interleukin 12p40 (IL-12p40), tumor necrosis factor-α (TNF-α) and interleukin 6 (IL-6) (Supplementary Fig. S2).

### Blood metabolomics

**Figure 2:**
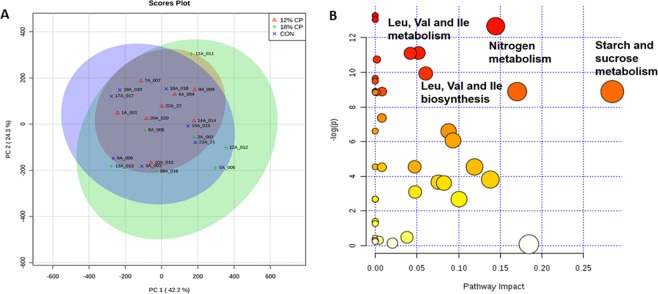
Principle component analysis (PCA) score plots and the pathway analysis map of serum metabolites in piglets fed with low protein diets. (**A**) PCA score plots of serum metabolites. Each node represents an individual pig. (**B**) The map of pathway analysis for the metabolites detected in the blood serum. Each circle represents a metabolic pathway with the scores obtained from topology analysis (pathway impact; the x-axis) and the pathway enrichment analysis (y-axis). The color of each circle is based on its *p*-value, while the size of each circle is based on its impact values. Therefore, larger size circles are indicative of higher pathway impact, while darker colors circles are suggestive of more significant changes of metabolites and higher pathway enrichment. No difference in pathway enrichment was found between CON and 18% crude protein (CP) or between 18% CP and 12% CP. Therefore, the data for both CON and 18% CP were combined and compared with 12% CP for pathway enrichment. CON, control diet with 24% CP; 18% CP, low protein diet with 18% CP; 12% CP, low protein diet with 12% CP. n = 7, n = 7, n = 8 for CON, 18% CP and 12% CP groups, respectively.

The principle component analysis (PCA) score plot for blood metabolites is shown in Fig. 2A. The CON diet was not clearly separated from the 18% CP and the 12% CP groups. The PC 1 is indicative of 42.2% variation in metabolite changes between samples and PC 2 explains 24.3% of the variation. Using the metabolic pathway enrichment analysis, nitrogen metabolism, starch and sucrose metabolism, and leucine, isoleucine and valine metabolism and biosynthesis were greatly influenced by the amount of protein in the diet (Fig. 2B).

The significantly different metabolites among dietary groups are shown in Table 2. The metabolites involved in nitrogen or protein metabolism, *i.e*. hydroxycarbamate N-acetylglutamate (NAG) and orotic acid were different among groups. As expected, amino acid profile and the metabolites involved with amino acid metabolism were changed significantly with modifying the dietary protein content (Table 2). Serum isoleucine, valine, tyrosine, tryptophan and glycine concentration were decreased in 12% CP compared to CON, whereas 12% CP had higher concentration of glutamic acid compared to 18% CP and CON. Further, 4-hydroxyhippuric acid and indole-3-acetate, were reduced in 12% CP group (P < 0.001 and *P* = 0.008, respectively). As indicated in pathway analysis, the starch and sucrose metabolism were influenced by dietary protein levels (Fig. 2B). The highest peak value for glucose was seen in 12% CP among all dietary groups (P < 0.001; Table 2). Of the lipid metabolites, serum cholesterol was higher in 12% CP compared to CON and 18% CP. Moreover, dietary groups showed differential changes in carbohydrates and amino acids derivatives and fatty acids and vitamins metabolites.

**Table 2:** Effect of dietary protein content on blood metabolomics profile.

Metabolites	CON^1,2^	18% CP^1,2^	12% CP^1,2^	SEM^3^	P-value
**Microbiome Metabolism**					
4-hydroxyhippuric acid	333.9^a^ ± 55.7	335.0^a^ ± 49.0	186.2^b^ ± 35.3	18.2	<0.001
Indole-3-acetate	1288.4^a^ ± 523.3	949.3^ab^ ± 257.9	776.2^b^ ± 217.7	85.5	0.008
**Carbohydrate Derivatives**					
Arabitol	9536.1^a^ ± 2895.9	12458.1^a^ ± 2777.1	26265.7^b^ ± 4795.0	1786.3	<0.001
Pinitol	6066.4^a^ ± 1439.3	5577.4^a^ ± 1678.6	1711.4^b^ ± 442.4	504.4	<0.001
Hexitol	3156.6^a^ ± 583.4	4568.1^a^ ± 1257.4	6908.6^b^ ± 1165.3	403.6	<0.001
Xylitol	4949.9^a^ ± 446.6	4736.9^a^ ± 890.5	3712.0^b^ ± 458.1	174.7	<0.001
Lactobionic Acid	2661.0^a^ ± 834.8	2188.4^ab^ ± 875.6	1246.9^b^ ± 611.8	204.1	<0.001
Conduritol-beta-epoxide	15246.9^a^ ± 6484.8	17466.1^a^ ± 8838.6	4229.9^b^ ± 1388.0	1802.1	<0.001
N-acetylmannosamine	325.9^a^ ± 161.6	561.4^ab^ ± 220.9	756.9^b^ ± 165.7	53.8	0.001
Saccharic acid	2087.3^a^ ± 719.1	2438.6^a^ ± 732.9	1391.9^b^ ± 288.8	155.7	0.002
**Carbohydrate Metabolism**					
UDP-glucuronic acid	593.6^a^ ± 152.8	485.6^a^ ± 91.4	309.7^b^ ± 108.2	35.5	<0.001
2-hydroxyglutaric acid	1026.1^a^ ± 300.2	1375.6^a^ ± 479.5	1910.5^b^ ± 380.4	113.2	0.001
Aconitic acid	1414.9^a^ ± 362.3	1233.7^ab^ ± 225.9	1024.9^b^ ± 231.5	66.5	0.002
Glucose-1-phosphate	1139.6^a^ ± 278.4	1368.1^ab^ ± 229.3	1719.6^b^ ± 273.8	74.9	0.008
**Carbohydrates**					
Glucose	60566.9^a^ ± 7699.4	109757.0^b^ ± 42503.7	147801.5^c^ ± 32350.6	10082.5	<0.001
**Protein Metabolism**					
Hydroxycarbamate	3102.6^a^ ± 1508.9	6206.6^a^ ± 2725.3	10706.1^b^ ± 2107.4	818.4	<0.001
N-acetylglutamate	221.1^a^ ± 90.1	223.7^a^ ± 94.3	381.6^b^ ± 74.7	24.2	0.003
Orotic acid	297.7^a^ ± 59.5	252.4^b^ ± 49.3	238.7^b^ ± 52.3	12.2	0.007
**Amino Acids**					
Isoleucine	311769.7^a^ ± 28746.2	292147.1^a^ ± 57318.2	189372.3^b^ ± 32707.5	14567.1	<0.001
Tryptophan	94968.1^a^ ± 31926.0	85616.6^a^ ± 26084.5	47116.6^b^ ± 8808.5	6667.3	<0.001
Oxoproline	818178.6^a^ ± 55568.7	712547.7^b^ ± 92182.9	643733.8^b^ ± 81095.4	22308.1	<0.001
Glutamic acid	43575.4^a^ ± 17906.9	62658.3^a^ ± 30741.2	103193.4^b^ ± 31564.9	7869.2	0.002
Glycine	670979.7^a^ ± 77092.1	622914.1^a^ ± 180096.8	414781.3^b^ ± 104649.7	35718.3	0.002
Valine	411978.9^a^ ± 49889.2	381391.3^ab^ ± 74580.3	294508.4^b^ ± 35679.9	15683.7	0.004
Tyrosine	341122.9^a^ ± 100236.5	311482.7^ab^ ± 43414.8	226762.3^b^ ± 39620.8	17163.5	0.004
**Fatty Acid Metabolites**					
Isohexonic acid	2325.6^a^ ± 275.0	2302.3^a^ ± 688.3	1412.5^b^ ± 338.3	133.5	<0.001
**Amino Acids Metabolism Metabolites**					
2-ketoisocaproic acid	17449.9^a^ ± 2218.4	14123.1^b^ ± 3060.5	11027.1^b^ ± 1627.7	747.8	<0.001
Aminomalonate	2319.7^a^ ± 1180.8	7096.4^b^ ± 3853.0	9997.9^b^ ± 3377.5	930.0	<0.001
Phenylethylamine	7534.6^a^ ± 4235.9	7324.0^a^ ± 2537.3	15673.0^b^ ± 3443.1	1116.0	<0.001
Putrescine	991.3^a^ ± 189.7	1671.3^b^ ± 395.3	2035.4^b^ ± 554.4	127.0	0.001
**Amino Acid Derivatives**					
Trans-4-hydroxy-L-proline	116148.9^a^ ± 18749.9	92179.4^b^ ± 16517.4	46283.7^c^ ± 21204.3	7496.0	<0.001
Pyrrole-2-carboxylic acid	2869.1^a^ ± 384.5	2591.3^ab^ ± 651.9	2025.7^b^ ± 323.9	122.8	0.003
N-acetylaspartic acid	1388.3^a^ ± 461.0	1300.1^a^ ± 673.8	718.9^b^ ± 156.6	115.7	0.003
**Vitamins**					
Dehydroascorbic acid	1446.7^a^ ± 430.3	2488.0^b^ ± 786.9	1334.2^b^ ± 477.9	162.9	0.002
**Lipid Metabolites**					
Cholesterol	275695.1^a^ ± 28637.7	305711.7^a^ ± 76128.6	396785.3^b^ ± 43574.5	15668.9	0.002

^1^CON, control diet with 24% crude protein (CP); 18% CP, low protein diet with 18% CP; 12% CP, low protein diet with 12% CP.

^2^Average peak height.

^3^SEM: standard errors of means.

^a,b^Within a row, values with different superscripts are different (P < 0.05). The values are the mean ± standard errors.

### Fecal microbiota

**Figure 3:**
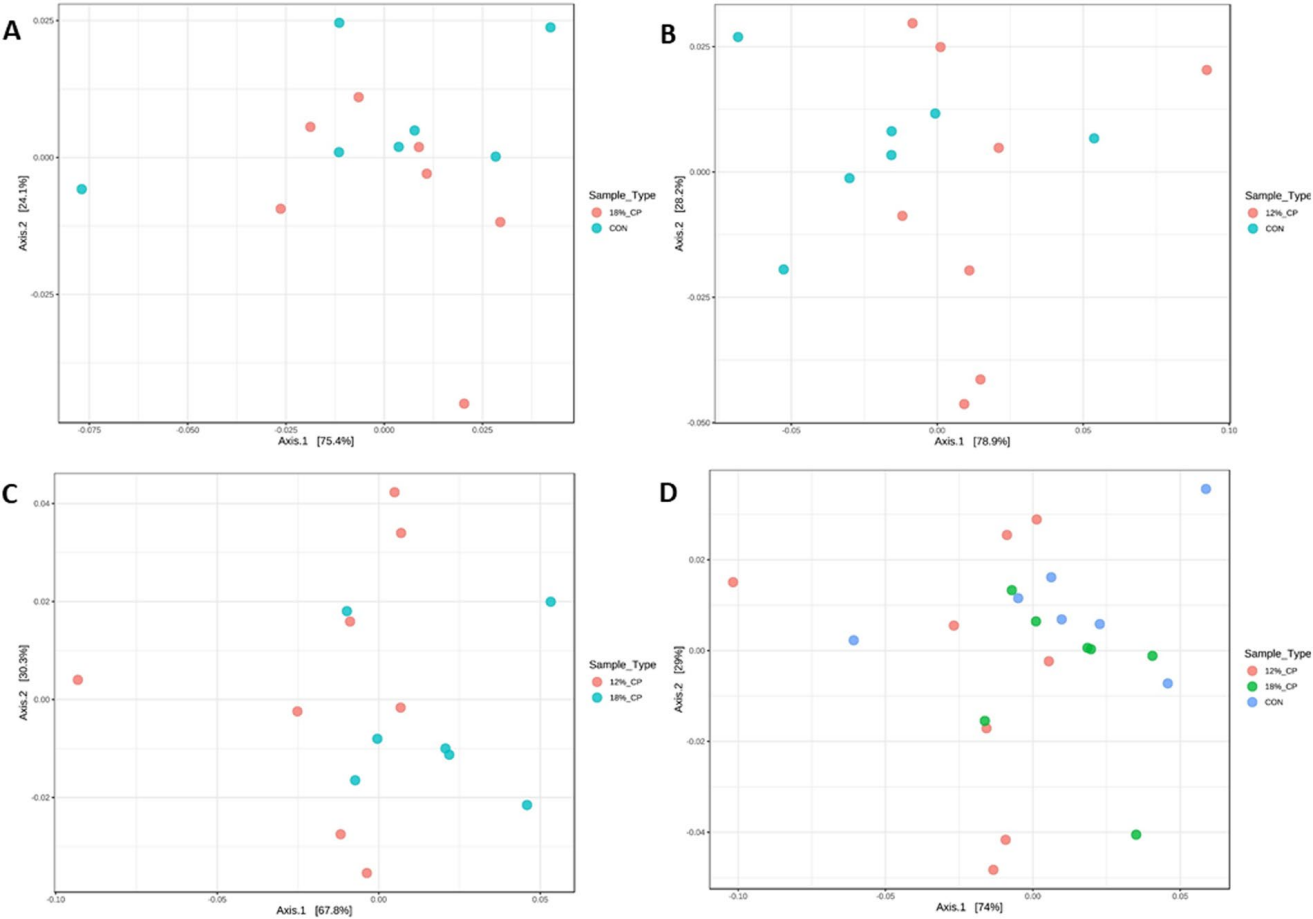
Beta diversity of the fecal bacterial community in nursery pigs fed with different levels of dietary protein at genus level. Principle coordinates analysis (PCoA) of fecal microbiota for (**A**) CON vs. 18% crude protein (CP), (**B**) CON vs. 12% CP, (**C**) 12% CP vs. 18% CP, (**D**) 12% CP vs. 18% CP vs. CON. Pigs are grouped based on their dietary treatments, *i.e*. CON, control diet with 24% CP; 18% CP, low protein diet with 18% CP; 12% CP, low protein diet with 12% CP. Each node represents an individual pig. Differences were considered significant at P < 0.05. The PERMANOVA P values for CON vs. 18% CP, CON vs. 12% CP, 12% CP vs. 18% CP and 12% CP vs. 18% CP vs. CON were 0.38, 0.07, 0.02 and 0.04, respectively. n = 7, n = 7, n = 8 for CON, 18% CP and 12% CP groups, respectively.

**Figure 4:**
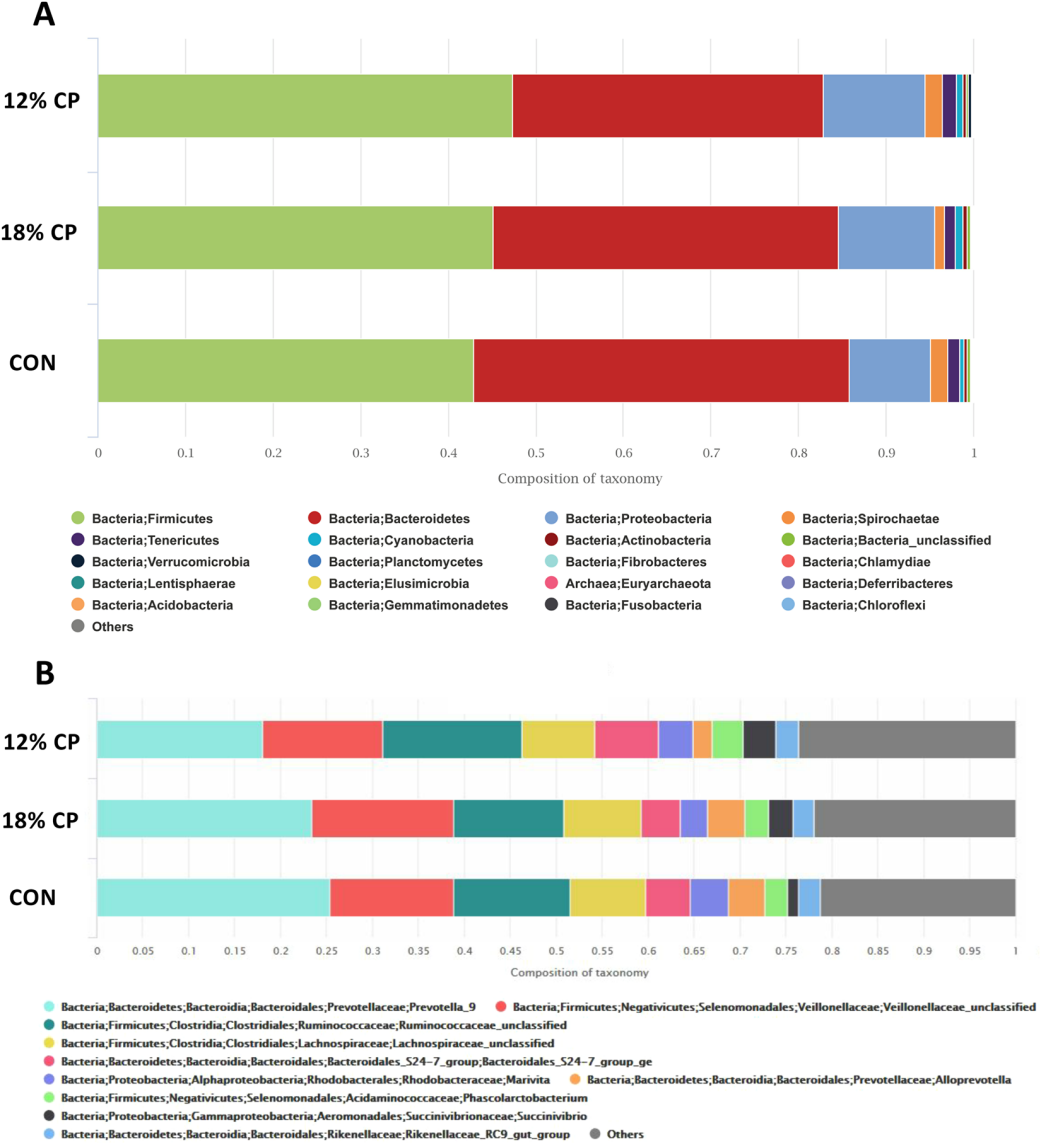
The effect of low protein diets on fecal bacterial community at phylum and genus level. (**A**) The relative abundance of bacterial community composition at phylum level in fecal samples of pigs fed with different levels of dietary protein. Only the top 20 phyla are depicted for clarity. (**B**) The relative abundance of bacterial community composition at genus level in fecal samples of pigs fed with different levels of dietary protein. Only the top 10 genera are depicted for clarity. CON, control diet with 24% crude protein (CP); 18% CP, low protein diet with 18% CP; 12% CP, low protein diet with 12% CP. n = 7, n = 7, n = 8 for CON, 18% CP and 12% CP groups, respectively.

Based on the rarefaction curve analysis, all samples analyzed reached a stable plateau at 40,000 reads and 1,000 operational taxonomic units (OTU’s) (Supplementary Fig. S3), which suggests the sequencing depth was sufficient to capture the species richness of the samples. Principle coordinates analysis (PCoA) showed a significant separation and clustering when 12% CP vs. 18% CP (PERMANOVA analysis P values = 0.02; Fig. 3C) and 12% CP vs. 18% CP vs. CON (PERMANOVA analysis P values = 0.04; Fig. 3D) were considered confirming the differences in gut microbiota composition among these dietary groups. No clear clustering was seen for fecal bacterial composition of CON vs. 12% CP (PERMANOVA analysis P values = 0.07; Fig. 3B) and CON vs. 18% CP groups (PERMANOVA analysis P values = 0.38; Fig. 3A). Overall, the three main phyla present in all three dietary treatments were Firmicutes, Bacteroidetes and Proteobacteria (Fig. 4A). The most abundant community at genus level for all three dietary treatments was *Prevotella* (Fig. 4B). At family level, Prevotellaceae, Veillonellaceae, Ruminococcaceae, and Lachnospiraceae were the most abundant communities across all diets (Fig. 4B).

**Figure 5:**
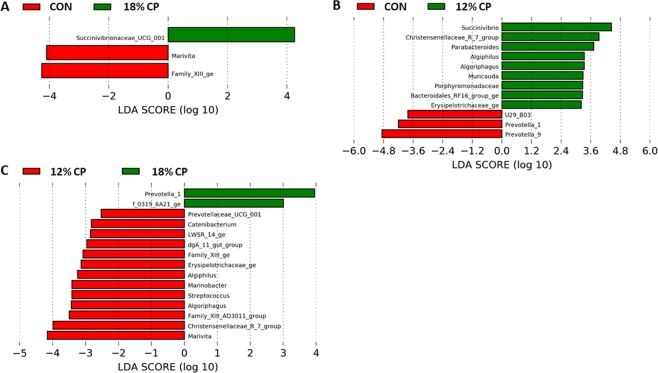
Effect of low protein diets on fecal microbiota composition at genus level using linear discriminant analysis (LDA) with effect size (LEfSe). (**A**) CON vs. 18% crude protein (CP), (**B**) CON vs. 12% CP, (**C**) 12% CP vs. 18% CP. CON, control diet with 24% CP; 18% CP, low protein diet with 18% CP; 12% CP, low protein diet with 12% CP. n = 7, n = 7, n = 8 for CON, 18% CP and 12% CP groups, respectively.

Linear discriminant analysis (LDA) with effect size measurements (LEfSe) was used to identify organisms that are different among dietary conditions and are also of statistical and biological significance. Compared to CON, 18% CP group had higher proportions of *Succinivibrionaceae_UCG_001* (LDA [log_10_] score> 2.0; Fig. 5A and Supplementary Fig. S4). Pigs fed with CON diet had higher proportions of *Prevotella_9* and *Prevotella_1* compared to those fed 12% CP diet (Fig. 5B and Supplementary Fig. S5). However, the feces of pigs fed 12% CP was enriched in *Succinvibrio, Christensenedilaceae_R_7_group*, *Parabacteroides, Algiphilus and Algoriphagus* compared to CON (Fig. 5B and Supplementary Fig. S5). Pigs fed with 12% CP diet had higher proportions of *Marivita*, *Christensenellaceae_R_7_group, Famuliy_XIII_AD3011_group, Algoriphagus, Streptococcus, Marinobacter and Algiphilus* than pigs fed 18% CP diet (Fig. 5C and Supplementary Fig. S6), while the fecal samples from 18% CP group had greater abundance of *Prevotella_1* (Fig. 5C and Supplementary Fig. S6).

## Discussion

The major criticism to modern swine production is related with excessive nitrogen excretion and pollution, which has adverse effects on the environment and human health. Moderate to severe reduction in dietary protein may reduce the nitrogen excretion from pigs; however, previous research provides evidence that reducing the dietary CP by more than 25% will depress their growth performance^11–15^. The underlying mechanisms by which low protein diets mediate the energy balance in weaned pigs is not well understood. Therefore, the objective of this study was to determine the effect of dietary CP level on growth performance and energy balance, metabolomicsprofile and fecal microbiota composition in young pigs. Our study revealed several important findings: 1) slight reduction in dietary protein (*i.e*. 18% CP) did not influence the energy balance measurements, but moderate reduction of dietary CP (*i.e*. 12% CP) decreased the feed intake and body weight and increased the EE in first week of the study. The increased EE together with decreased feed intake contributed to reduced growth performance of pigs fed with moderately low protein diets, 2) decreasing the dietary CP by 50% (*i.e*. 12% CP) influenced the metabolism of branched-chain amino acids (BCAA), metabolites of amino acids, nitrogen and protein, lipids and vitamins metabolites, carbohydrates and microbiome metabolites, 3) pigs fed with moderately low protein diets had higher abundance of *Christensenedilaceae, Algoriphagus and Algiphilus* and lower abundance of *Prevotella* in their feces compared to those fed with slightly low protein or control diets. Further, feces of pigs fed with low protein diets was more enriched in Succinivibrionaceae family than those in control group. Overall, reducing the dietary CP level by 25% (*i.e*. 18% CP) did not significantly influence the growth performance, health and metabolism; however, reducing the dietary CP by 50% decreased the growth performance and impacted gut microbiota and blood metabolites.

Reduction of dietary protein by 25% produced no significant effects on body weight and feed efficiency, but moderate reduction of dietary protein decreased the body weight. These results are consistent with previous studies conducted in young to finisher pigs^11–15^. Little is known on the underlying mechanisms for differential metabolic responses to slightly low and moderately low protein diets in young pigs. The animals fed with 12% CP diet, but not 18% CP diet, reduced feed intake and increased EE during early weeks of study, which could contribute to depressed growth performance in that group. The data on the effect of low protein diets on EE of pigs is scarce and inconsistent. Pigs fed with 12% CP had increased EE during the first week. In support of this finding, other pioneer studies using limited number of pigs reported increased EE in growing or young pigs fed with severely low protein diets^24–27^. Inconsistent with our data, other studies reported no effect^28,29^ or a linear decrease^10,30,31^ in heat production when dietary CP was decreased moderately in growing and finishing pigs. The discrepancy in EE data among different studies can be attributed to variation in age of animals used, duration of  EE measurement and micronutrient composition of the diets. Clearly, more research is needed to understand the effects of dietary protein on EE in pigs.

To our best knowledge, there is no published research on the effect of low protein diets on blood metabolomics profile in nursery pigs. Metabolic pathway analysis of blood serum metabolites showed that starch and sucrose metabolism were the key metabolic pathways influenced by the level of dietary protein in nursery pigs. Pigs fed with 12% CP diet had higher serum glucose and glucose-1-phosphate and had lower serum uridine diphosphate glucuronic acid (UDP- glucuronic acid), a key intermediate involved in synthesis of essential glycoconjugates, which can be synthesized from UDP-glucose, the precursor of glycogenesis^41^. Increased glucose-1-phosphate and decreased UDP- glucuronic acid in serum of pigs fed with 12% CP diet is suggestive of changes in glycogen metabolism. Low protein diets were shown to increase the rate of glycogenesis and concentration of hepatic glycogen in rats^42^. Also, pigs fed 12% CP diet increased the level of serum 2-hydroxyglutaric acid, which then can be converted to alpha-ketoglutarate through the action of 2-hydroxyglutarate dehydrogenase^43^. Alpha-ketoglutarate is one of the key mediators of tricarboxylic acid cycle acid (TCA) that determines the overall rate of TCA^44^. Variation in serum metabolites level such as 2-hydroxyglutaric, aconitic, fumaric and succinic acids (shown in Table 2 and raw metabolomics data) in response to diets with variable protein contents might be suggestive of alteration in TCA cycle rate of pigs fed with these diets. Further research is required to assess the activity of key rate-limiting enzymes regulating the rate of TCA cycle such as alpha-ketoglutarate dehydrogenase in pigs fed with low protein diets.

The metabolic pathway analysis of serum metabolites further revealed that the nitrogen metabolism pathway as well as BCAA metabolism and biosynthesis were significantly impacted by the amount of dietary CP. The 12% CP increased the serum NAG, an essential activator cofactor of carbamoyl‐phosphate synthetase 1 (CPS1), which catalyzes the synthesis of carbamoyl‐phosphate, a key rate limiting enzyme in urea synthesis. This is suggestive of increased activity of NAG synthase in pigs fed with very low protein diets, which catalyzes the synthesis NAG from glutamate. Increased NAG in response to very low protein diet in the current study may not be essentially interpreted as the increased rate of ureagenesis as low protein diets provide less NH3 as the substrate for synthesis of CPS-1 and in general are recommended for patients with urea cycle disorders to prevent hyperammonemia^45^. Low protein diets decreased the serum orotic acid, a compound which is synthesized from carbamoyl-phosphate when ornithine transcarbanoylase (OTC) enzyme is deficient or unable to handle the carbamoyl-phosphate load within the urea cycle to convert it to citrulline^46^. The decreased concentration of serum orotic acid in low protein groups is suggestive of highly functional OTC likely due to less abundance of nitrogen and carbamoyl-phosphate entering to urea cycle. As expected, the concentration of serum isoleucine and valine as well as 2-ketoisocaproic acid, an intermediate of leucine metabolism were decreased in pigs received moderately low protein diets. This is in agreement with other studies where a decrease in serum BCAA concentration was reported by decreasing the level of dietary protein in growing pigs^47^. Indole-3-acetate, which is produced by *Bacteroides, Clostridia, and E. coli* via catabolizing tryptophan in small intestine^48,49^ and intermediate produced from BCAA by *Enterobacter cloacae*^50^, were decreased in pigs fed with moderately low protein diets, which is suggestive of reduced availability of tryptophan and BCAA as a substrate for bacterial use. Further, the pigs fed with diets with 12% CP had lower concentration of isohexonic acid. Branched-chain fatty acids are produced exclusively by fermentation of proteins in the gut^51^. Similar to our data, others showed a decreased level of isobutyrate, isovalerate, and branched-chain fatty acids in cecal contents of growing pigs fed with low protein diets^39^. The decreased serum branched-chain fatty acids concentration is most likely due to less protein available for the gut bacteria for production of these fatty acids. Pigs fed with moderately low protein-high carbohydrate diets had the highest serum cholesterol concentration. This is while low protein-high carbohydrate diets have been related with better cardiometabolic health and life span in rodent studies^52–54^. Given the positive link between the blood cholesterol concentration and atherosclerosis in humans^55^ and the similarities between pigs and humans in terms of nutritional physiology^56–58^, further research is need to delineate the cholesterol metabolism in humans when they consume low protein-high carbohydrate diets.

The data on the gut microbiota composition of nursery pigs fed with various levels of dietary protein is scarce. The three main phyla in the feces of all dietary treatments were Firmicutes, Bacteroidetes and Proteobacteria. Similarly, other studies reported Firmicutes and Bacteroidetes as the main phyla in feces and cecal digesta of pre and post weaned and growing pigs^59–61^. In finishing pigs, Firmicutes, Proteobacteria and Actinobacteria represented the majority of bacterial communities in ileal samples^38,61^, but in colonic contents Firmicutes, Bacteroidetes, and Spirochaetae were the most abundant populations^38^. It is well established that with advancing the age of weaned piglets the composition of gut microbiome changes^37^. At genus level, the most abundant bacterial community for all three dietary treatments was *Prevotella* and at family level Prevotellaceae, Veillonellaceae, Ruminococcaceae, and Lachnospiraceae were the most dominant communities in the current study. In line with our data, others showed that healthy nursery pigs had a high abundance of Prevotellaceae, Rumbinococcaceae and Lactobacillaceae in their feces^62^. In addition, like our data others showed that after weaning, there is an increased abundance of *Prevotella* in the gut microbiota, which is believed to be increased due to their ability to degrade plant-based feed that contain hemicelluloses and xylans^59,63–65^.

In the current study, the fecal samples collected from pigs fed with moderately low protein diet were less enriched in *Prevotella*. The lower percentage of hemicellulose and likely its component, xylan^66^ in moderately low protein diets, which serve as a substrate for *Prevotella*^67,68^ may explain the low numbers of this bacteria in feces of pigs fed with those diets in the present study. The moderately low protein diets, although had the highest carbohydrate%, their hemicellulose content was the lowest (0.46%, 0.57% and 0.69% for 12% CP, 18% CP and CON groups, respectively) due to their decreased soybean meal content, which is contributing as the major source of hemicellulose for diets in the present study. The high abundance of *Prevotella* has been linked with increased body weight and growth rate in weaned pigs^69^ and this could be due to their role in the metabolism of complex polysaccharides^70^. Therefore, the lower abundance *Prevotella* in the gastrointestinal tract of pigs received moderately low protein diet may partly explain their poor performance in the present study. Our data is not in parallel with previous research showing higher abundance of *Prevotella* in colonic contents of weaned pigs fed with low protein (14% and 17% CP) diets^71^ or in cecal content of growing pigs fed with diets with reduced protein content (15% CP)^39^. The discrepancy in data may be attributed to the sources of carbohydrates used as well the dietary protein content in different studies. Further, for the first time we report here that moderately low protein diets increased the fecal proportions of *Christensenedilaceae, Algoriphagus and Algiphilus* in weaned pig model. In studies conducted both in human and rats, the increased abundance of *Christensenedilaceae* was linked to lower body mass index^72–74^. The association of *Algoriphagus and Algiphilus* with dietary protein content is remained to be studied. In the present study, pigs received low protein diets had more enriched Succinivibrionaceae family in feces, which might be due to high concentration of starch and non-fiber carbohydrates in their diets that are used as substrate for Succinivibrionaceae family^75,76^. Pigs fed with moderately low protein diets had higher abundance of fecal *Parabacteroides* and *Porphyromonadaceae* compared to those fed with standard protein diet. The high abundance of *Parabacteroides* has been linked with weight loss^77^. Treating ob/ob and high-fat diet fed mice with live *Parabacteroides distasonis* decreased the weight gain and hyperglycemia^77^. Similarly, the decreased levels of gut *Porphyromonadaceae* is associated with weight gain and obesity in rats^78^. Thus, the higher gut *Parabacteroides* and *Porphyromonadaceae* might contribute to reduced growth of pigs fed with moderately low protein diets. The pigs fed with slightly low protein diets had lower abundance of *Family_XIII_ge* and *Marivita* in their feces relative to those fed with standard protein or moderately low protein diets. The association of the above-mentioned bacteria with dietary proteins and their role in metabolic responses to dietary protein content have yet to be investigated.

A potential caveat with our study is that we did not collect the fecal samples in the beginning of study and before starting our experimental diets to report longitudinal microbiota data. Therefore, one may argue that the differences in microbiota composition seen among groups may have been present from the beginning of study due to effect of environmental factors on microbiota composition. However, it is noteworthy that the housing (*e.g*. pen size, location of study, etc.), source of animals, husbandry (*e.g*. lighting, room temperature, feeding frequency, etc.), duration of adaption and experimental periods, and other practical aspects of the study were identical for dietary groups and hence the contribution of those factors on the differences observed in fecal microbiota composition of different dietary groups would be negligible in this study. Further, in the present study the experimental low protein diets used had higher concentration of carbohydrates relative to control diet; therefore, the observed responses such as reduced *Prevotella* in fecal samples of pigs fed with these diets and etc. may be the result of either low dietary protein or changes in content and form of carbohydrates.

With current environmental concerns related with nitrogen excretion and pollutions from modern pig production and its consequences on human health, providing pigs with diets with markedly reduced protein content has been suggested as one of dietary strategies to decrease the nitrogen excretion^3^. However, the main limitation in applying moderate to severe reduction in dietary protein content is its subsequent negative influence on growth performance of pigs, which is discouraging to implement at commercial swine industry levels. Our data shed light on the mechanisms by which low protein diets influence the growth performance of pigs suggesting decreased feed intake, increased EE and differential changes in plasma metabolites and gut microbiota composition as few explanations. Here we show that metabolism of BCAA are highly impacted with variation in dietary protein content suggestive of a need for further evaluation of the effect of low protein diets supplemented with leucine, isoleucine and valine as next limiting amino acids on energy intake and expenditure. We also report a smaller number of *Prevotella* in feces of pigs fed with moderately low protein diet which may contribute to poor performance of these pigs^69^. This may suggest examining the role of specific dietary probiotics and phytogenic compounds that can potentially change the gut microbiota and influence growth performance and energy balance of pigs received very low protein diets. Characterizing the combination of individual amino acids and other dietary additives supplemented in low protein diets for wean-to-finisher pigs that will improve feed efficiency and limit nutrient excretion and emission of volatile compounds to the environment would contribute not only to sustainability of swine production worldwide, but also to safe environment for humans.

## Conclusions

In summary, feeding nursery pigs with moderately low protein diets resulted in reduction in feed intake, body weight and gain:feed ratio, but increased the energy expenditure, while feeding them with slightly low protein diets did not influence growth performance. The pigs fed with low protein or standard protein diets showed differential bacterial communities in their feces as well as serum metabolomics profile. The low protein diet influenced the sucrose and starch metabolism, nitrogen metabolism and branched-chain amino acids metabolism and biosynthesis pathways. The feces of pigs fed with moderately low protein diet was less enriched in *Prevotella*, but had higher proportions of *Christensenedilaceae*, *Aligiphilus* and *Algoriphagus*.

## Methods

### Animals, housing and experimental diets

All methods were performed in accordance with the relevant guidelines and regulations of Animal Care and Use Committee at Oklahoma State University and the experimental procedures were approved by this committee (Animal Care and Use Protocol – AG-17-27). A total of 37 crossbred (PIC^®^) pigs were used. Pigs were weaned on 21 days of age and were given a 14 days of adaptation period in a large pen in the same room. The pigs were housed in an environmentally temperature and ventilation-controlled facility.

Following 14 days of adaption period, the pigs were weight-matched (8.41 ± 0.14 kg), and individually housed in the same room that they were acclimated. Following individual housing, the animals were randomly assigned into three dietary treatments with different levels of dietary protein content for 28 days: control (24% CP; CON; n = 12), slightly low protein (18% CP; n = 12) and moderately low protein (12% CP; n = 13). The ingredients and composition of the experimental diets are shown in Table 3. The diets were formulated to meet the requirements for 7–11 kg pigs as recommended by National Research Council (NRC)^79^. The CON diet was formulated based on NRC standard ileal digestibility (SID) recommendation for amino acids, which came to be equivalent to 24% CP. For the remaining two dietary groups, the CP content of CON diet was reduced by 25% and 50% resulting in 18% and 12% CP, respectively. All diets were isocaloric, *i.e*. consistent metabolizable energy (ME) and consistent in crude fat content (ME = 1.5 MCal/Kg and crude fat = 3.6%; Table 3). The amounts of soybean meal as the major source of protein and corn and corn starch as the sources of carbohydrate were manipulated in order to achieve the desired protein levels while maintaining the energy content consistent throughout all dietary treatments (Table 3). In other words, the corn and cornstarch were added at the expense of soybean meal in low protein diets. The dietary carbohydrate percentage was 70.5%, 76.2% and 81.7% for CON, 18% CP and 12% CP diets, respectively. All remaining nutrients were kept consistent among diets and added at or above the requirements listed in the NRC^79^. The pigs had *ad libitum* access to feed and water, throughout the study. Animals were fed once every day at ~ 1500.

**Table 3:** Composition of experimental diets (as-fed basis).

Ingredients, %	CON^1^	18% CP^1^	12% CP^1^
Corn, yellow dent	52.60	64.63	76.09
Fish meal, menhaden	5.00	5.00	5.00
Soybean meal, 47.5% CP	34.50	18.52	3.94
Whey, dried	6.00	6.00	6.00
Corn starch	—	3.65	6.29
Dicalcium phosphate 18.5%	0.65	1.06	1.44
Limestone	0.59	0.50	0.55
Salt	0.26	0.25	0.27
Vitamin premix^2^	0.25	0.25	0.25
Trace mineral premix^3^	0.15	0.15	0.15
**Chemical Composition**			
Crude protein, %			
Calculated^4^	24.00	18.00	12.00
Analyzed	23.00	19.00	12.00
SID Lysine, %^4^	1.32	0.91	0.54
SID Threonine, %^4^	0.83	0.60	0.40
SID Methionine, %^4^	0.38	0.30	0.23
SID Tryptophan, %^4^	0.27	0.18	0.1
Crude Fat, %			
Calculated^4^	3.61	3.60	3.61
Analyzed	3.10	2.70	3.20
Carbohydrate, %	70.49	76.19	81.73
ME, Mcal/kg^5^			
Calculated^4^	1.5	1.5	1.5

^1^CON, control diet with 24% crude protein (CP); 18% CP, low protein diet with 18% CP; 12% CP, low protein diet with 12% CP.

^2^Vitamin mix provided per kilogram of diet: vitamin A, 720,000 IU; vitamin D, 180,000 IU; vitamin E, 5,400 mg; vitamin K3, 360 mg; vitamin B12, 3.15 mg; niacin, 6,750 mg; pantothenic acid, 2,250 mg; riboflavin, 675 mg; phytase, 61,200 FTU.

^3^Mineral mix provided per kilogram of diet: copper sulfate, 11,000 ppm; iodine, 198 ppm; iron, 73,413 ppm; manganese, 22,046; selenium, 198 ppm; zinc, 73,413 ppm.

^4^Values were calculated using National Swine Nutrition Guide (NSNG; V 2.0) software; standard ileal digestibility (SID).

^5^Metabolizable energy (ME).

### Metabolic measurements

Individual feed intake was measured daily and body weight was recorded weekly throughout the study. ADG, ADFI, G:F, ADPI, G:P and weekly feed intake, body weight, G:F and G:P were then calculated.

Daily EE and RQ was measured using indirect calorimetry system (AEI Technologies, Chicago, IL). Due to limitation in number of chambers (total 6), 22 pigs from CON, 18% CP and 12% CP groups (n = 7, n = 7, n = 8, respectively) were rotated for EE measurement every 48 h throughout the study by transferring them into the calorimetry chambers from their individual pens. The pigs had free access to feed and water in each chamber. The EE for each pig was measured from 0800 h to 1500 h, with the total measurement of 7 h. The O2 and CO2 sensors were calibrated by known volume of O2 (16% and 21%) and CO2 (0.03% and 4%) prior to each test every day. To allow the system to stabilize, the data for first 2 h were excluded from statistical analysis. For measuring the daily total EE, flow rate was set at 4–20 L/min depending upon the weight of pigs during the experiment with a sampling time of 5 seconds/chamber, stabilization period of 55 seconds and a reference air measurement after every 3 chambers. The O2 consumption rate (VO2 ml/min), CO2 production rate (VCO2 ml/min) and the RQ (VCO2/VO2) were measured. The normalized EE [(kcal/h)/kg body weight ^0.75^] was calculated using the following formula: [3.815 + (1.232 × RQ )] × VO2 (L/h)^80^.

### Feed, fecal and blood samples collection

Feed samples (~50 g) were collected during the diet preparations from each feed bag and pooled for each diet. The samples were then stored at −20 °C until proximate analysis for feed composition. Fecal samples (n = 7, n = 7, n = 8 for CON, 18% CP and 12% CP groups, respectively) were collected on day 42 of the study. Fresh fecal samples were collected from the rectum of all pigs, placed in a pre-labeled 50 mL falcon tubes (VWR Radnor, PA) on ice, transported to the lab and stored at −80 °C for further analysis for DNA extraction and microbiota composition.

Approximately 10 ml of blood (n = 7, n = 7, n = 8 for CON, 18% CP and 12% CP groups, respectively) was collected from the jugular anterior vena cava in the supine position in sterile dry tubes (BD Vacutainer^®^, Franklin Lakes, NJ) on day 42. The blood samples were placed on ice immediately after collection, transferred to the lab, centrifuged at 2,000 x g for 10 min at 4 °C and serum was separated. The collected serum was stored at −80 °C until further analysis for cytokines concentration and metabolomics profile.

### Diet composition analysis

Diet composition analyses were performed by Servi-Tech laboratories (Dodge City, KS). Experimental diets were analyzed for moisture and dry matter^81^, CP^82^, crude fat^83^, crude fiber^84^, calcium and phosphorus^85^ and nitrogen^86^.

### Serum cytokines analysis

 Using an enzyme-linked immunosorbent assay kit (R&D Systems, Inc., Minneapolis, MN), serum samples were analyzed in duplicate according to the manufacturer’s instructions for TNF-α, IL-6 and IL-8 concentrations. The absorbance values were measured using a microplate reader (Spectramax M3®; Molecular Devices, LLC, San Jose, CA) at 450 nm with the correction wavelength set at 570 nm. The intraassay CV for TNF-α, IL-8 and IL-6 was 4.7%, 2.6% and 16.0%, respectively. Using the Luminex™ (Thermo Fisher Scientific, Inc., Waltham, MA) multi-analyte profiling technology, a bead-based assay (ProcartaPlex™ multiplex immunoassay) was used to determine the concentration of a panel of cytokines including IL-12p40 according to manufacturer’s protocol. The intraassay CV for IL-12p40 was 9.3%.

### Serum metabolomics

Sample preparation, data acquisition and data processing were performed at West Coast Metabolomics Center (UC Davis, Davis, CA) as previously described^87,88^. Briefly, following sample preparation using a mixture of acetonitrile:isopropanol:water (3:3:2, *v*/*v*/*v*) as well as 1:1 acetonitrile:water for removal of protein from serum, the samples were dried down in a CentriVap concentrator. Then methoxyamine hydrochloride in pyridine was added to each sample for derivatization. The samples were then analyzed by gas chromatography (GC)- mass spectrometry (MS) using a time of flight mass spectrometer (Leco Pegasus IV). For data acquisition, a GC (Agilent 690) equipped with automated liner exchange (ALEX; Gerstel corporation) and cold injection system (CIS; Gerstel corporation) was used. The CIS temperature was set at 50 °C to 250 °C final temperature at a rate of 12 °C s^−1^. Injection volume was 0.5 µL and injection mode was splitless with a purge time of 25 s. Following every 10 injections, the injection liner was changed and before and after each run, the injection syringe was washed with 10 µL of ethyl acetate. A Rtx-5Sil MS column (30 m length x 0.25 mm internal diameter with 0.25 μm film made of 95% dimethyl/5%diphenylpolysiloxane; Restek corporation) protected by a 10 m long empty guard column was used. Mobile phase was Helium with a flow rate of 1 mL min^−1^ and column temperature was 50–330 °C. The GC temperature program was: 50 °C for 1 min, then ramped at 20 °C min^−1^ to 330 °C and held constant for 5 min. The transfer line temperature was set to 230 °C between GC and MS. Mass spectrometer with unit mass resolution at 17 spectra s^−1^ with a scan mass range of 80–500 Dalton was used. Electron ionization at −70 V and 1800 V detector voltage was employed with a temperature set at 250 °C for ion source. After data acquisition, raw GC-TOF MS data files were preprocessed with ChromaTOF vs. 2.32 and apex masses were reported for use in BinBase algorithm as previously described^87,88^. Result *.txt files were further processed after exporting to a data server with absolute spectra intensities by filtering algorithm employed in the metabolomics BinBase database. Quantification values were reported as peak height for the quantification ion (mz value) at the specific retention index, which is more precise than peak area for low abundant metabolites. All database entries that were positively detected in more than 10% of the samples of a study design class for unidentified metabolites were reported.

### Fecal microbiome

Fecal DNA was isolated using the QIAamp DNA stool mini kit (Qiagen, Inc., Germantown, MD) per manufacturer’s instructions. A Nanodrop spectrophotometer (Nanodrop® Technologies, Wilmington, DE) was used for quantification of the DNA concentration as well as its purity of the samples. Isolated DNA samples were stored at −80 °C until analysis. The samples with DNA concentration greater than 7 ng/ul were used for PCR amplification and 16 S amplicon sequencing at Novogene (Novogene, Corp., Sacramento, CA). Briefly, 16 S rRNA V4 region was amplified by PCR using the following primers: 515F (5′-GTGCCAGCMGCCGCGGTAA-3′) and 806R (5′-GGACTACHVGGGTWTCTAAT-3′) and Phusion® High-Fidelity PCR Mater Mix (New England Biolabs, Ipswich, MA, USA). PCR products were mixed with identical volume of 1X loading buffer containing SYBR green and loaded on 2% electrophoresis agarose gel for quantification and quality control. Using Qiagen Gel Extraction Kit (Qiagen, Hilden, Germany), the PCR products were extracted from the agarose gel and then the sequencing library was prepared (TruSeq DNA PCR-Free Sample Preparation Kit; Illumina, San Diego, CA) following the manufacturer’s instructions. Index codes were added and the library quality was determined by using the Qubit 2.0 Fluorometer (Thermo Scientific, Waltham, MA) and Agilent Bioanalyzer 2100 system. Using Illumina HiSeq. 2500 platform (Illumina, Inc., San Diego, CA), the library was sequenced and 250 bp paired-end reads were generated.

Sequence data analysis was performed by mothur (v. 1.39.5) using the mothur MiSeq standard operating procedure^89^. Briefly, paired-end reads were assembled and assigned to each sample based on their unique barcode. Contigs that were longer than 275 bp, or contained more than 8 homopolymers, or contained undetermined base were excluded. The trimmed sequences were then aligned against the SILVA-based V4 reference alignment, further denoised and subjected to chimera removal using Uchime^90^. Then, using the Bayesian classifier and Silva non-redundant database v132 as the reference^91^, the sequences were classified. After exclusion of Archaea, chloroplasts, mitochondria, and eukaryotic sequences, the remaining sequences were assigned to OTUs based on at least 97% similarity. These OTUs were then classified into taxonomic groups at a threshold of 80%.

### Statistical analysis

The daily and weekly measurements were analyzed using a general linear mixed model (IBM SPSS Statistics Version 23, Armonk, NY, USA). Diet, time and the interaction of diet and time were included in the model as fixed effects and the pig was considered as random variable. There was no effect of gender in the model, therefore it was excluded from the model. Based on the smallest values of fit statistics for corrected Akaike’s Information Criterion and Bayesian Information Criterion, the covariance structure of the repeated measurements for each variable was modeled as either first-order antedependence, autoregressive, heterogenous autoregressive, compound symmetry, heterogenous compound symmetry or toeplitz. The ADG, G:F, ADFI, ADPI and G:P and blood cytokines data were analyzed using univariate GLM procedure (IBM SPSS Statistics Version 23, Armonk, NY, USA). Means between dietary treatments were separated by Tukey’s post hoc test for all data. Differences were considered significant at P < 0.05 and a trend at 0.05 < P < 0.10.

The metabolomics data were analyzed using MetaboAnalyst 3.0^92^, which is available online at: http://www.metaboanalyst.ca/faces/ModuleView.xhtml. Data was filtered using interquantile range and the peak height was normalized to the median of all samples. A one-way ANOVA was performed to determine significantly different metabolites, within the serum, and false discovery rate was applied (not shown in the manuscript) to indicate the expected false positives among metabolites that are significant. Furthermore, Tukey’s post hoc test was used to determine significant mean changes between diets for each metabolite. Differences were considered significant at P < 0.05 and a trend at 0.05 < P < 0.10. A PCA was performed to differentiate the metabolites found in each diet, using the same program^92^. A pathway impact analysis was performed to determine the effects of dietary CP level on the metabolic pathways and metabolite enrichment. No difference in pathway enrichment was found between CON and 18% CP or between 18% CP and 12% CP. Therefore, the data for both CON and 18% CP were considered “high protein” and the data for 12% CP was considered “low protein” and analyzed for pathway enrichment. For microbiota data, the BIOM file containing taxonomic classifications of OTUs generated using Mothur was uploaded to a web-based data visualization tool, MicrobiomeAnalyst^93^ (https://www.microbiomeanalyst.ca/) to generate beta diversity plots at genus level and PERMANOVA statistics. The ordination method was selected as PCoA and Janson-Shannon divergence was used for distance mode in MicrobiomeAnalyst. To determine the differences in gut microbiota composition among dietary groups, LDA with LEfSe was performed at the genus level using a tool hosted in the Galaxy (server) instance of Huttenhower lab (https://huttenhower.sph.harvard.edu/galaxy/) and the scores were normalized by log10^94^. The bacteria with LDA score (log10)> 2 was considered as populations with distinctly increased numbers. To measure the effect size of differentially abundant taxons, Kruskal–Wallis and pairwise Wilcoxon tests were performed with differences being considered significant at P value < 0.05.

## Supplementary information

Supplementary Information.

## Data Availability

The sequencing data for microbiota and raw metabolomics data generated during the current study are  publically available in the National Center for Biotechnology Information (NCBI) Sequence Read Archive (SRA) with BioSample accession number SAMN13069906, [http://www.ncbi.nlm.nih.gov/bioproject/578706] and MetaboLights with the identifier MTBLS1316 [https://www.ebi.ac.uk/metabolights/MTBLS1316] repositories, respectively.
