# Supplementary material for: Low Protein-High Carbohydrate Diets Alter Energy Balance, Gut Microbiota Composition and Blood Metabolomics Profile in Young Pigs
Source: Sci Rep. 2020 Feb 24;10:3318. doi: 10.1038/s41598-020-60150-y (PMC7040010; doi:10.1038/s41598-020-60150-y)
Supplement: Supplementary file 1 — Supplementary Information. [file 41598_2020_60150_MOESM1_ESM.pdf]

Supplementary Information

Low Protein-High Carbohydrate Diets Alter Energy Balance, Gut Microbiota Composition and Blood Metabolomics Profile in Young Pigs

Shelby Spring[1], Hasitha Premathilake[1], Udaya DeSilva[1], Cedrick Shili[1], Scott Carter[1] and Adel Pezeshki[1*]

[1]Department of Animal and Food Sciences, Oklahoma State University, Stillwater, OK 74078, USA

*Correspondence author:

Adel Pezeshki, PhD

Department of Animal and Food Sciences

206C Animal Science Building

Oklahoma State University

Stillwater, OK 74078, USA

Phone: (405) 780-2464

E-mail: adel.pezeshki@okstate.edu

Supplementary Figures

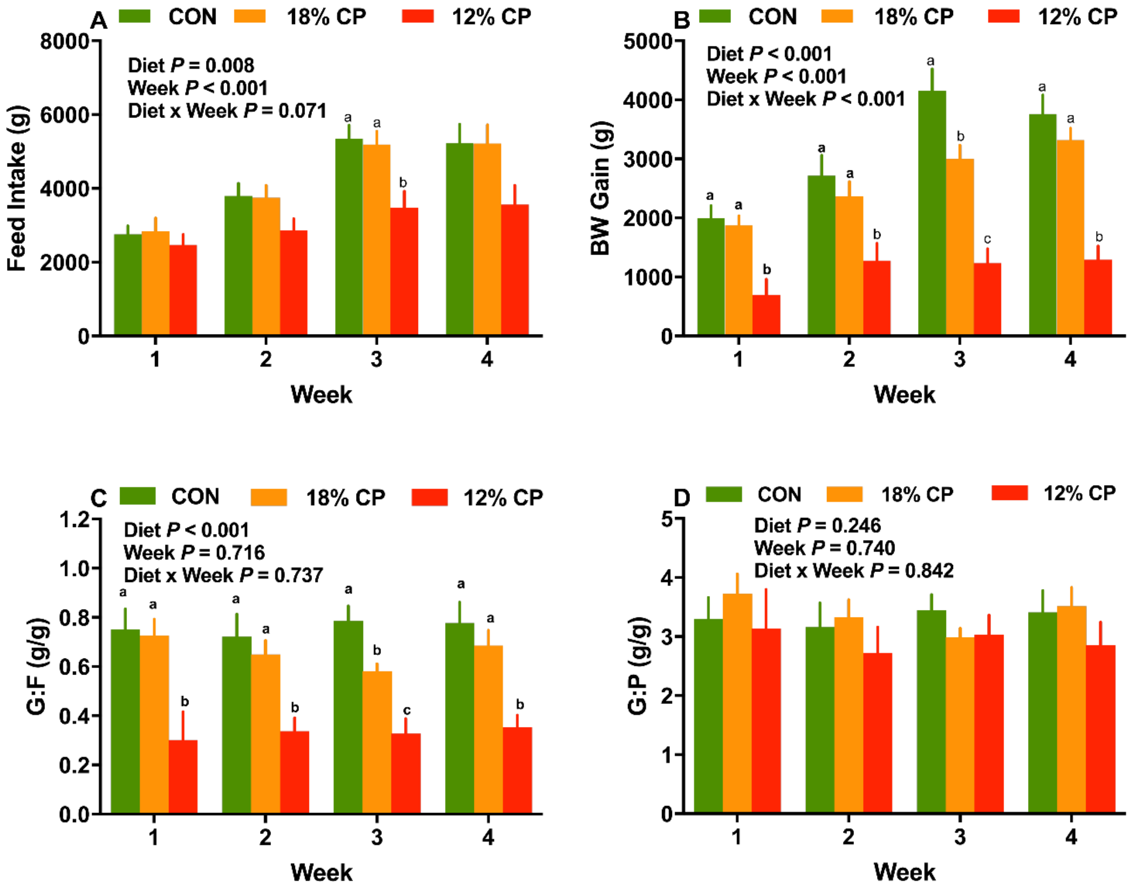

Supplementary Fig. S1: The effect of low protein diets on weekly feed intake, body weight gain and feed and protein efficiency.

(**A**) cumulative feed intake, (**B**) body weight (BW) gain, (**C**) gain: feed (G:F), (**D**) gain: protein (G:P). CON, control diet with 24% crude protein (CP); 18% CP, low protein diet with 18% CP; 12% CP, low protein diet with 12% CP. Among groups, values with different superscripts are significantly different ($P < 0.05$). The values are means ± standard errors of means, n=12, n=12, n=13 for CON, 18% CP and 12% CP groups, respectively

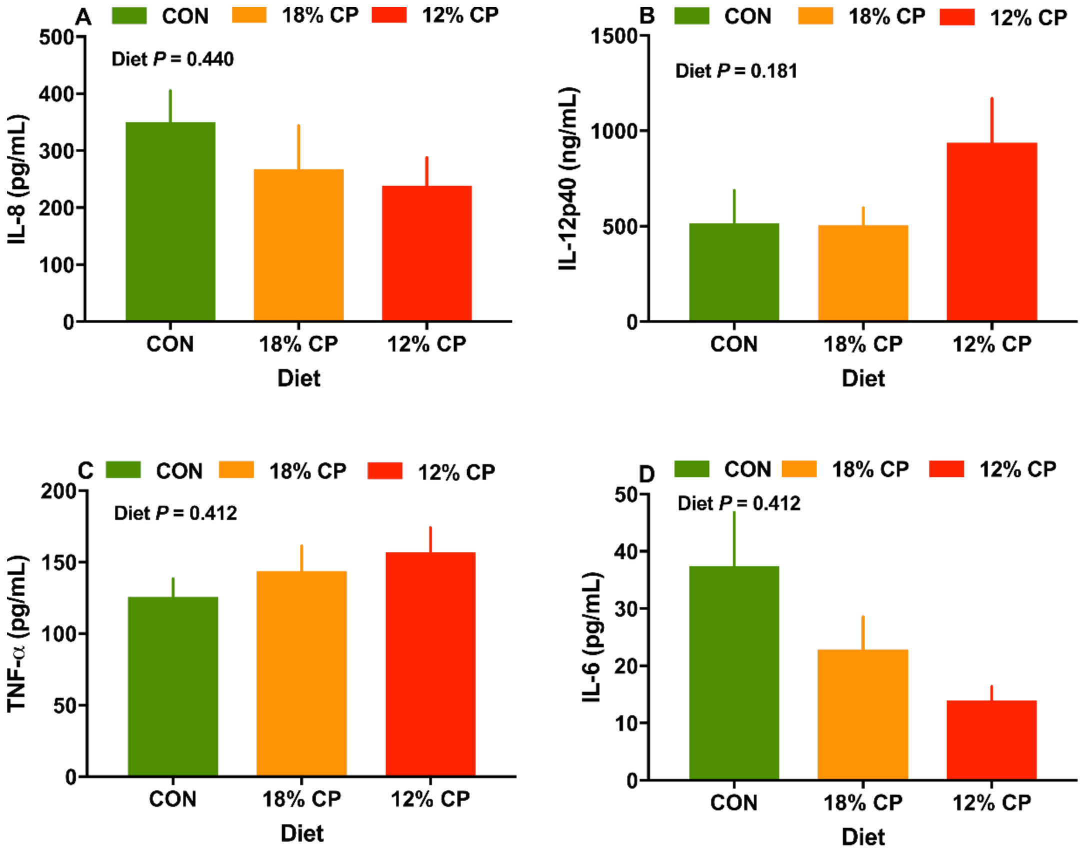

Supplementary Fig. S2: The effect of low protein diets on the concentration of serum cytokines.

(**A**) interleukin 8 (IL-8), (**B**) interleukin 12p40 (IL-12p40), (**C**) tumor necrosis factor-α (TNF-α), (**D**) interleukin 6 (IL-6). Values lower than the kit's sensitivity were replaced with the second lowest concentration of the standard curve. CON, control diet with 24% crude protein (CP); 18% CP, low protein diet with 18% CP; 12% CP, low protein diet with 12% CP. The values are means ± SEM, n=7, n=7, n=8 for CON, 18% CP and 12% CP groups, respectively.

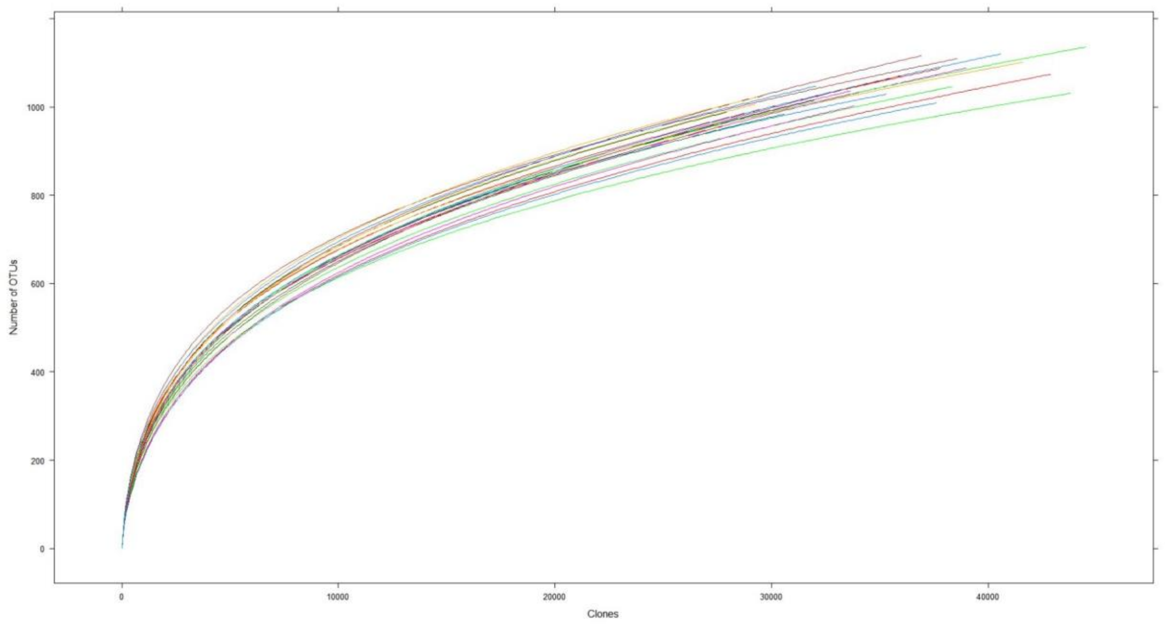

Supplementary Fig. S3: Rarefaction curve analysis for fecal samples collected from pigs fed with three dietary treatments including control diet (CON) with 24% crude protein (CP); low protein diet with 18% CP and low protein diet with 12% CP.

The rarefaction curves show the number of operational taxonomic units (OTU's) found as a function of the number of reads sampled. Each line represents an individual pig. n=7, n=7, n=8 for CON, 18% CP and 12% CP groups, respectively

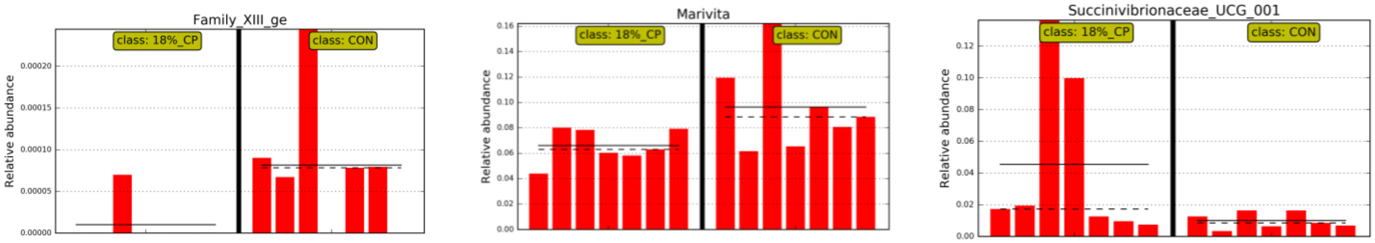

Supplementary Fig. S4: The relative abundance of bacterial communities in feces of pigs fed with control (CON) and 18% crude protein (CP) diets using linear discriminant analysis (LDA) with effect size (LEfSe).

The horizontal lines denote the group means, and the dotted lines mean the group medians. Each bar represents an individual pig. CON, control diet with 24% CP; 18% CP, low protein diet with 18% CP. n=7 for CON and n=7 for 18% CP groups.

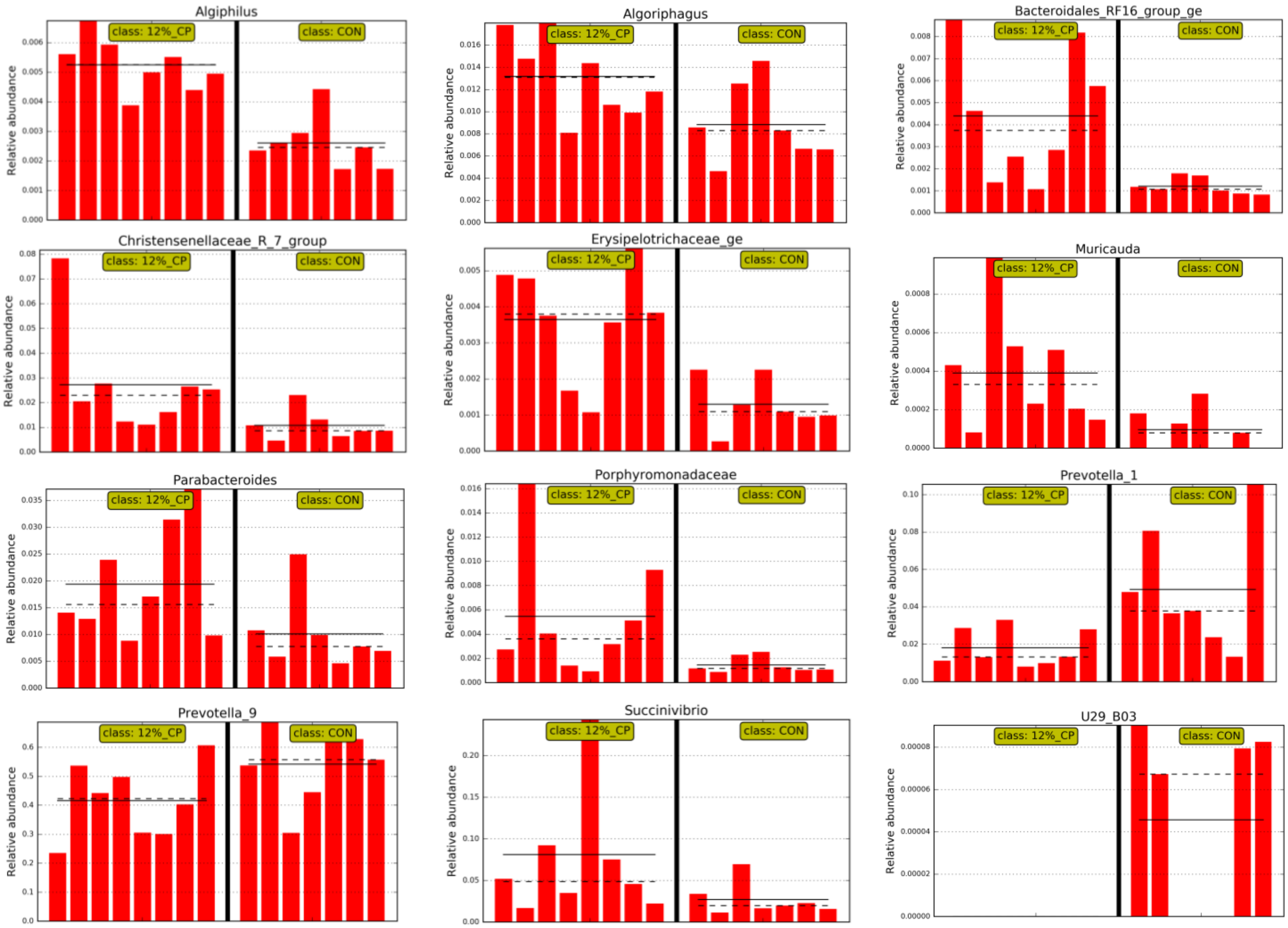

Supplementary Fig. S5: The relative abundance of bacterial communities in feces of pigs fed with 12% crude protein (CP) and control (CON) diets using linear discriminant analysis (LDA) with effect size (LEfSe).

The horizontal lines denote the group means, and the dotted lines mean the group medians. Each bar represents an individual pig. CON, control diet with 24% CP; 12% CP, low protein diet with 12% CP. n=7 for CON and n=8 for 12% CP groups.

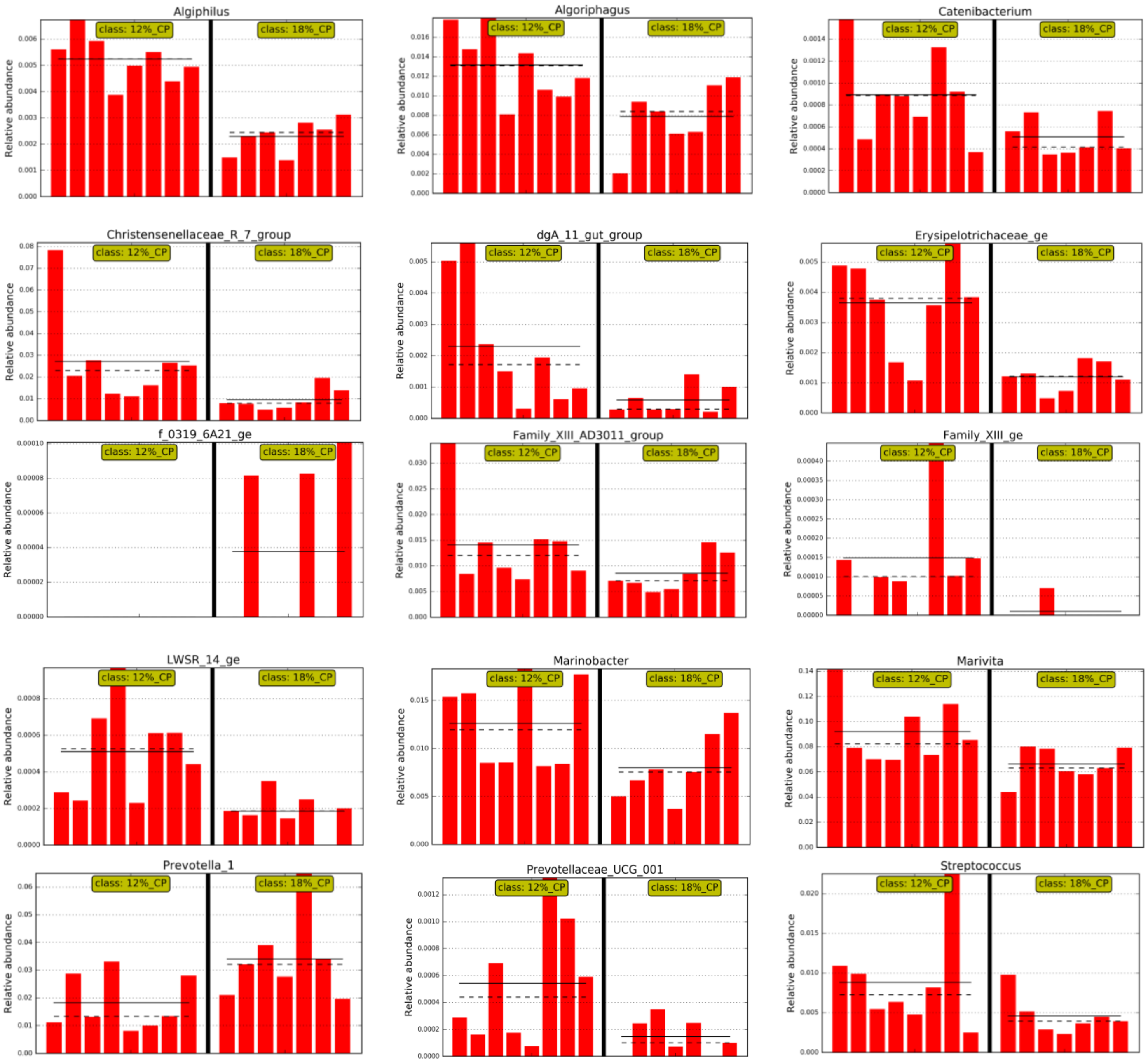

Supplementary Fig. S6: The relative abundance of bacterial communities in feces of pigs within 12% crude protein (CP) and 18% CP groups using linear discriminant analysis (LDA) with effect size (LEfSe).

The horizontal lines denote the group means, and the dotted lines mean the group medians. Each bar represents an individual pig. 18% CP, low protein diet with 18% CP; 12% CP, low protein diet with 12% CP. n=7 for 18% CP and n=8 for 12% CP groups.
